# Genetic diversity and population structure of *Haemonchus contortus* isolates from sheep across Xinjiang, China

**DOI:** 10.1017/S0031182025101352

**Published:** 2026-02

**Authors:** Waresi Tuersong, Reyilanmu Tuerhong, Abudusaimaiti Tuoheti, Ailixire Maimaiti, Dilare Xuekelaiti, Lianxi Xin, Aiheda Aideli, Yan Xiao, Min Hu, Saifuding Abula, Chahan Bayin, Qingyong Guo, Wei Zhang

**Affiliations:** 1 College of Veterinary Medicine, Xinjiang Agricultural University, Urumqi, XJ, China; 2 College of Veterinary Medicine, Xinjiang Agricultural University, Xinjiang Key Laboratory of New Drug Research and Development for Herbivorous, Urumqi, XJ, China; 3 College of Veterinary Medicine, Veterinary Medicine Postdoctoral Research Station of Xinjiang Agricultural University, Urumqi, XJ, China; 4 Veterinary Research Institute, Xinjiang Academy of Animal Husbandry, Urumqi, XJ, China; 5 National Key Laboratory of Agricultural Microbiology, College of Veterinary Medicine, Huazhong Agricultural University, Wuhan, China

**Keywords:** anthelmintic resistance, genetic diversity, *Haemonchus contortus*, mitochondrial *nad4* gene, population structure, Xinjiang

## Abstract

*Haemonchus contortus*, a highly pathogenic gastrointestinal nematode, significantly impacts small ruminant production, causing substantial economic losses in sheep and goat farming. This study examined the genetic diversity and population structure of 171 *H. contortus* isolates collected from the abomasa of sheep slaughtered across 8 distinct regions in Xinjiang, China. Using sequence analysis, phylogenetic reconstruction and population genetic analyses of the mitochondrial *nad4* gene, we identified 163 haplotypes, with haplotype diversity ranging from 0.995 to 1.000 and nucleotide diversity from 0.02007 to 0.03145. The Tacheng population displayed the highest nucleotide diversity. Analysis of molecular variance revealed that 91.83% of genetic variation occurred within populations, with minimal differentiation among them (Fst: −0.01296 to 0.04274). Neutrality tests (Tajima’s D and Fu’s Fs) indicated no recent population bottlenecks. Phylogenetic and haplotype network analyses showed no distinct geographic clustering, suggesting extensive gene flow, likely facilitated by host movement. These findings provide critical insights into the genetic structure of *H. contortus* in Xinjiang, informing strategies for managing anthelmintic resistance and controlling this economically significant parasite.

## Graphical abstract

**Figure:**
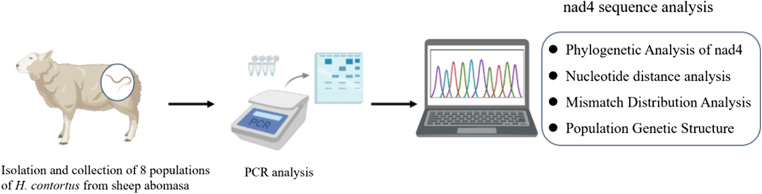

## Introduction

Xinjiang, a key hub for animal husbandry in China, boasts vast grasslands that support extensive livestock production (Zheng and Qian, 2003). Sheep and cattle farming are vital to the region’s economy (Dawut and Tian, 2021). However, livestock diseases, particularly parasitic infections, pose significant threats to animal health and farm profitability (Yan, 2022). Among these, *Haemonchus contortus*, a highly pathogenic gastrointestinal nematode, is a major constraint to ruminant production (Adduci et al., 2022). This parasite infects the abomasa of domestic ruminants, including sheep, goats and cattle, as well as wild ruminants, and occasionally the small intestine (Besier et al., 2016; Arsenopoulos et al., 2024). Its blood-feeding behaviour causes anaemia, diarrhoea, weight loss and, in severe cases, death (Adduci et al., 2022). Seasonal reactivation of diapause larvae, known as the ‘spring rise’, triggers disease outbreaks, leading to significant livestock mortality (Jianhua and Longxian, 2013). Consequently, *H. contortus* contributes to substantial economic losses globally, with estimated annual treatment costs of USD 26 million in Kenya, USD 46 million in South Africa and USD 103 million in India (McRae et al., 2014; Palevich et al., 2019; Memon et al., 2024).

Located in central Eurasia along the Silk Road, Xinjiang’s strategic position facilitates extensive ruminant trade with mainland China and neighbouring countries. The region’s diverse terrain and climate, ranging from cold high-altitude zones to warm lowlands, influence parasite survival and distribution. Human activities, such as livestock movement, and environmental factors, including climate change, drive genetic diversity in *H. contortus*, with evidence of selection pressures from anthelmintic use and local climatic variations (Sallé et al., 2019).

In China, *H. contortus* severely impacts animal husbandry, particularly sheep farming, across most provinces (Yang et al., 2016; Youwei et al., 2021; Ting, 2024; Zhiya et al., 2024). Despite global studies on *H. contortus* genetic diversity in regions such as Europe, the USA, Brazil, Australia, Malaysia and China, no data exist for Xinjiang (Yin et al., 2013; Shen et al., 2017; Pitaksakulrat et al., 2021). This study addresses this gap by analysing the genetic diversity and population structure of 171 *H. contortus* individuals from 8 populations across southwestern, central and northeastern Xinjiang using the mitochondrial *nad4* gene. These findings aim to elucidate the genetic characteristics of *H. contortus* in Xinjiang, providing a foundation for developing targeted, region-specific strategies to manage anthelmintic resistance and control this economically significant parasite.

## Materials and methods

### Parasite material

**Figure 1.**
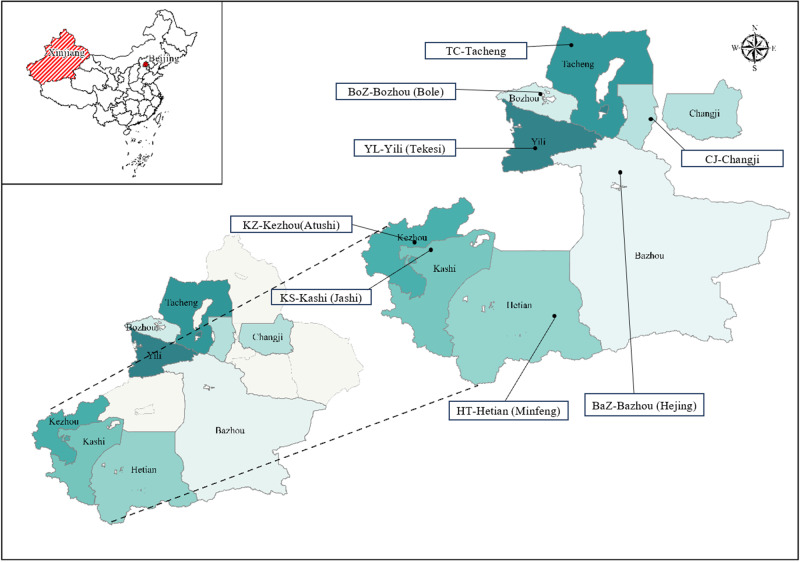
Sampling sites. Eight different geographical locations in China (longitudes and latitudes given in Table 1) at which adult *Haemonchus contortus* were collected from sheep.

A total of 171 adult *H. contortus* worms were collected from the abomasa of sheep slaughtered at 8 locations across southern and northern Xinjiang, China, including Bazhou (Hejing), Kezhou (Atushi), Kashi (Jashi) and Hetian (Minfeng) in southern Xinjiang, and Yili (Tekesi), Tacheng, Changji and Bozhou in northern Xinjiang (Table 1 and Figure 1). These locations were separated by 550–2000 km. Worms were washed thoroughly in physiological saline, preserved in 70% ethanol and transported to the Parasitology Laboratory, College of Veterinary Medicine, Xinjiang Agricultural University, Urumqi. Upon arrival, worms were identified based on morphological characteristics, including the twisted digestive and reproductive tracts and copulatory bursa, following Tuerhong et al. (2025). Specimens were stored at −20 °C until further analysis.

**Table 1.** Collection sites for sheep-derived *H. contortus* samples across 8 regions in Xinjiang, China

Region	Geographic coordinates	Sample
Kezhou (Atushi)	39.73°N, 76.17°E	24
Kashi (Jashi)	39.71°N, 76.18°E	21
Bazhou (Hejing)	42°19′19.1″N, 86°22′54.3″E	23
Hetian (Minfeng)	37.13°N, 80.73°E	20
Yili (Tekesi)	43.1°N, 80.30°E	24
Tacheng	46.75°N, 82.98°E	21
Changji	44.02°N, 87.29°E	22
Bozhou (Bole)	44.9°N, 80.27°E	16

### Genomic DNA isolation

Genomic DNA was extracted from adult male *H. contortus* worms using the Tiangen Genomic DNA Extraction Kit (DP-304, Tiangen Biotech, Beijing, China), following the protocol outlined in Tuerhong et al. (2025). DNA concentrations were quantified using a NanoDrop 2000 spectrophotometer. Samples with concentrations exceeding 30 ng μL^−1^ were stored at −20 °C until further analysis.

### PCR amplification and sequencing

The ITS-2 region (265 bp) and mitochondrial *nad4* gene (800 bp) of 171 *H. contortus* were amplified by polymerase chain reaction (PCR) using specific primer sets and thermal cycling conditions (Table 2; Bott et al., 2009; Yin et al., 2013). Each PCR reaction was conducted in a 25 µL volume, containing 2.0 µL of template DNA, 1.0 µL of forward primer, 1.0 µL of reverse primer, 12.5 µL of 2× TransStart FastPfu Fly PCR SuperMix (TransGen Biotech, Beijing, China) and 8.5 µL of DEPC-treated water. Post-amplification, 5 µL of PCR products were analysed by 1.0% agarose gel electrophoresis to confirm target amplicons. Amplicons were sequenced (Sanger) by Wuhan Qingke Biotechnology Co., Ltd (Wuhan, China).

**Table 2.** Primers and thermal cycling conditions for PCR amplification of *H. contortus ITS-2* and *nad4* genes

Target gene	Primer	Sequence (5′-3′)	PCR protocol
*ITS-2*	NC1-F	CAAATGGCATTTGTCTTTAG	94 °C for 5 min; 35 cycles of 94 °C for 30 s, 55 °C for 30 s, 72 °C for 30 s; 72 °C for 7 min
	NC1-R	TTAGTTTCTTTTCCTCCGCT	
*nad4*	Primer1-F	GGATTTGGTCAGCAAATTGAA	94 °C for 5 min; 35 cycles of 94 °C for 30 s, 55 °C for 30 s, 72 °C for 1 min; 72 °C for 10 min
	Primer2-R	GCCTGCAAATGAATTAACA	

### Data analysis

Sequencing results were aligned against NCBI databases to confirm the acquisition of target *ITS-2* and *nad4* gene sequences. Sequences were aligned using Clustal W in MEGA 7.0 (Tamura et al., 2004) and manually trimmed. Homology comparisons were performed using BioEdit v7.2 (Tippmann, 2004) between the obtained sequences and reference sequences from GenBank (accession numbers: MK300723.1, KF176320.1, PP531612.1, and MW662167.1). Haplotype diversity, nucleotide diversity and neutrality tests (Tajima’s D and Fu’s Fs) were calculated using DnaSP v6.0 (Rozas et al., 2017). Haplotype sequences were aligned in MEGA 7.0 using *Haemonchus placei* (GenBank accession: AF070825.1) as the out-group, and a phylogenetic tree was constructed using the neighbour-joining (NJ) method with 1,000 bootstrap replicates. AMOVA and genetic differentiation (Fst) were conducted using Arlequin v3.5 (Excoffier et al., 1992). A haplotype network was generated using PopART v1.7 (Leigh et al., 2015).

## Results

### Sequences analyses

**Figure 2.**
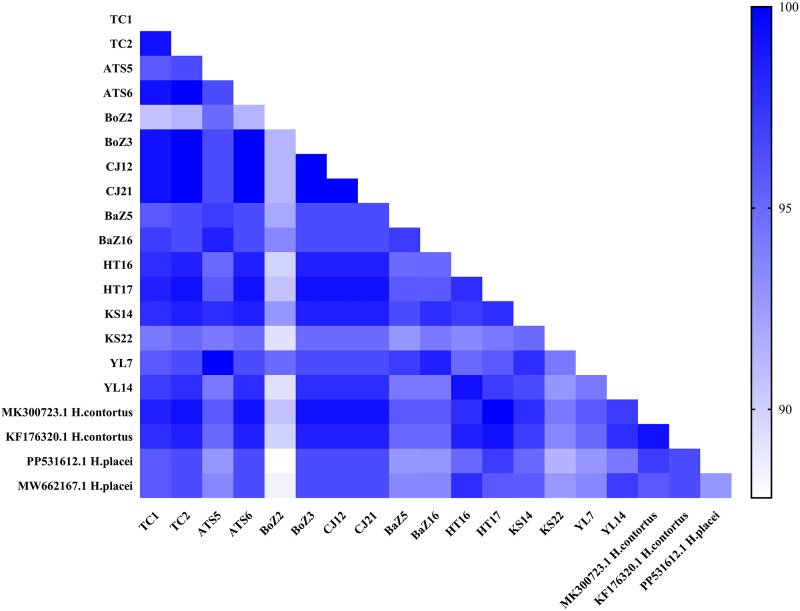
Pairwise sequence homology (%) of 16 *ITS-2* sequences from *H. contortus* with reference sequences of *H. contortus* and *H. placei* from GenBank.

To further validate the species identity, a homology analysis was performed on a representative subset of 171 sequenced samples. These samples had previously been aligned and confirmed as *H. contortus* using NCBI. Specifically, 2 sequences were randomly selected from each of the 8 geographical regions (16 sequences in total) and aligned against reference sequences. These included 2 *H. contortus* reference sequences (GenBank: MK300723.1 and KF176320.1) and 2 *H. placei* reference sequences (GenBank: PP531612.1 and MW662167.1). Sequence homology with *H. contortus* ranged from 92.8% to 100%, while nucleotide identity with *H. placei* ranged from 87.8% to 97.8% (Figure 2). These alignments confirmed that all sequenced samples were *H. contortus*. The majority of these sequences exhibited high sequence similarity (≥99%) with *H. contortus* reference sequences, consistent with the established molecular identification threshold for this genus (Stevenson et al., 1995). However, 2 sequences (e.g., from samples HT16 and YL14) displayed intermediate similarity to both reference species. Subsequent inspection of their sequencing chromatograms revealed heterozygous base calls at diagnostic nucleotide positions, a pattern indicative of interspecific hybridization between *H. contortus* and *H. placei*, as previously reported (Brasil et al., 2012). With the exception of these 2 putative hybrids, the alignments confirmed the identity of the remaining sequenced samples as *H. contortus*.

Population genetic analysis, based on the *nad4* gene, was therefore conducted on a confirmed dataset of 171 pure *H. contortus* samples. This analysis identified 163 haplotypes, with 16–24 haplotypes per population. Nucleotide diversity across the 8 populations ranged from 0.02007 to 0.03145, with the Tacheng population exhibiting the highest value (0.03145). Haplotype diversity ranged from 0.995 to 1.000, reflecting high genetic variation. Tajima’s D values ranged from −1.711 to −0.862, and Fu’s Fs values ranged from −19.345 to −7.669 (Table 3). These findings suggest no recent population bottlenecks in the 8 populations.

**Table 3.** Genetic diversity parameters of the *nad4* gene in *H. contortus* populations from 8 regions in Xinjiang, China

Region	Sequences (*n*)	*H*	Pi	Hd	*K*	Tajima’s D	Fu’s Fs
CJ	22	22	0.02626	1.000	14.913	−1.10132	−10.999
TC	21	20	0.03145	0.995	17.862	−1.28536	−6.270
BoZ	16	16	0.02582	1.000	14.667	−0.86194	−6.246
YL	24	23	0.02836	0.996	16.109	−1.12145	−9.231
HT	20	19	0.02879	0.995	16.353	−0.92392	−6.102
KS	21	21	0.02007	1.000	11.362	−1.71111	−12.346
BaZ	23	23	0.02651	1.000	15.059	−1.24827	−11.820
ATS	24	24	0.02187	1.000	12.424	−1.24724	−14.635
Total	171	163		0.9994			

### Phylogenetic analysis of *nad4*

**Figure 3.**
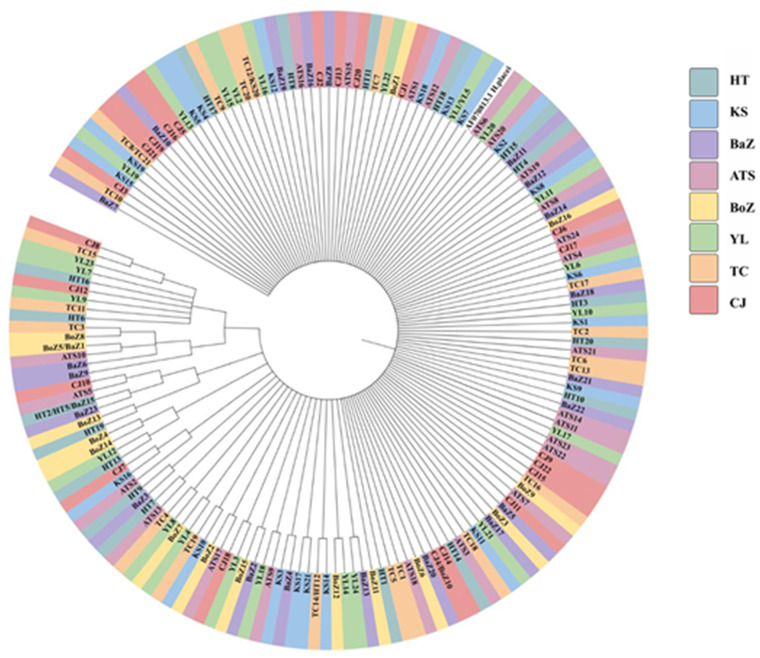
Phylogenetic tree of 163 *nad4* haplotypes from *H. contortus* constructed using the neighbour-joining (NJ) method.. TC: Tacheng, ATS: Atushi, YL: Yili, KS: Kashi, HT: Hetian, HJ: Hejing, CJ: Changji, BoZ: Bozhou.

A phylogenetic tree was constructed using *nad4* sequences from 163 *H. contortus* isolates from Xinjiang, China, with *H. placei* (GenBank: AF070825.1) as the out-group (Figure 3). Genetic distances among haplotypes showed continuous variation, with sequences distributed across diverse geographic regions without clear geographic clustering. Most nodes had low bootstrap support (<55%), but a clade of 2 Atushi isolates, 1 Hetian isolate and 1 Yili isolate showed moderate support (71%). Additionally, a clade comprising 2 Bozhou isolates, 1 Changji isolate, 1 Bazhou isolate and 2 Yili isolates exhibited strong bootstrap support (91–96%).

**Figure 4.**
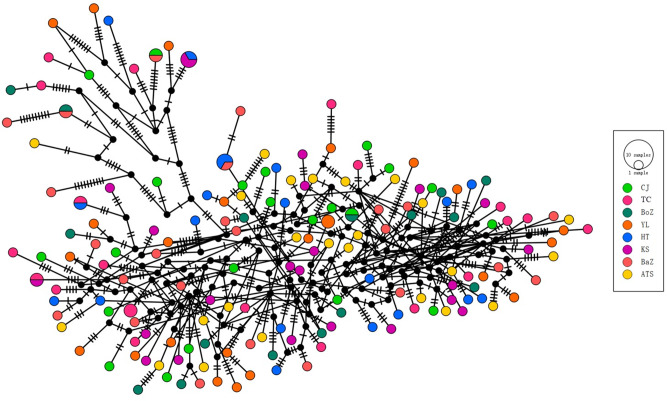
Haplotype network of 163 *nad4* haplotypes from *H. contortus* across 8 regions in Xinjiang, China.

To further explore genetic relationships among the 163 *nad4* haplotypes, a haplotype network was constructed (Figure 4). Results revealed no distinct geographic clustering of haplotypes, consistent with phylogenetic analysis. However, a few haplotypes from Bazhou and Yili formed relatively distinct branches, corroborating the phylogenetic findings.

### Nucleotide distance analysis

**Figure 5.**
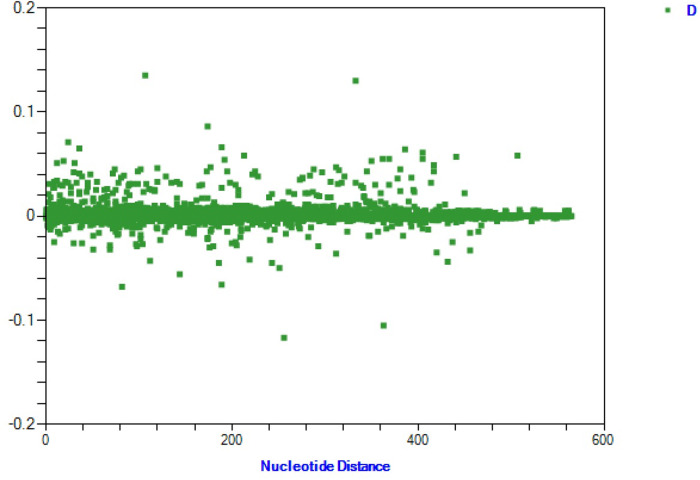
Genetic distance analysis of *nad4* sequences from 8 *H. contortus* populations in Xinjiang, China.

Nucleotide distance analysis of 8 *H. contortus* populations from Xinjiang, China, revealed minimal variation in genotype frequencies and no significant population structure (Figure 5). These results align with genetic differentiation (Fst) and analysis of molecular variance (AMOVA), both indicating high gene flow among populations. Geographic isolation did not significantly influence genetic differentiation, with no evidence of subpopulation structure driven by geographic distance. Thus, frequent gene flow results in a largely homogeneous genetic structure across populations.

### Mismatch distribution analysis

**Figure 6.**
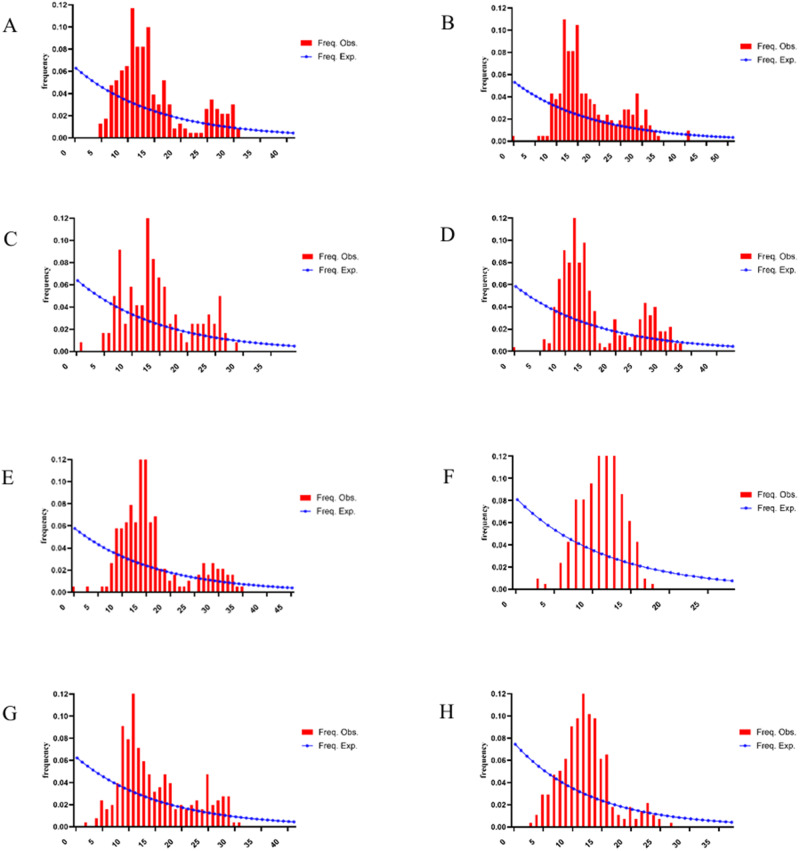
Mismatch distribution of *nad4* gene sequences from 8 *H. contortus* populations in Xinjiang, China. A:CJ.B:TC.C:BoZ.D:YL.E:HT.F:KS.G:BaZ.H:KeZ.

Mismatch distribution analysis of the *nad4* gene in 8 *H. contortus* populations from Xinjiang, China, revealed a multimodal distribution (Figure 6). The red curve represents the expected distribution under a population expansion model, while the blue bars depict the observed distribution from sequencing data. A unimodal distribution typically indicates recent population expansion, whereas a multimodal distribution suggests population stability. The multimodal pattern observed across Xinjiang populations, together with significantly negative Fu’s Fs values and evidence of high gene flow, indicates a structured population experiencing demographic expansion.

### Population genetic structure

**Figure 7.**
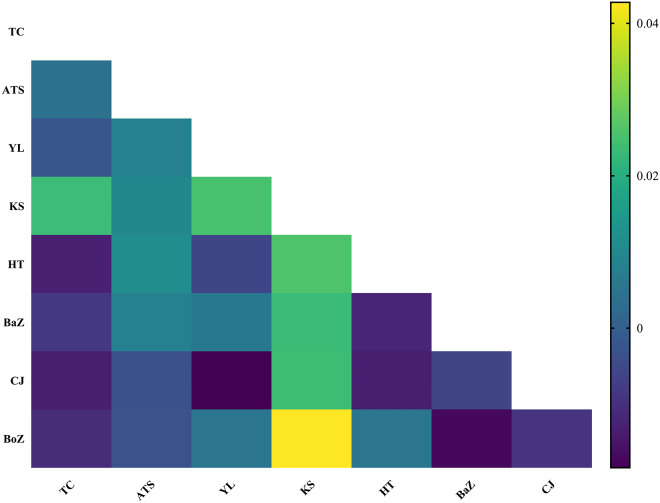
Pairwise Fst values among 8 *H. contortus* populations from Xinjiang, China. TC: Tacheng, ATS: Atushi, YL: Yili, KS: Kashi, HT: Hetian, HJ: Hejing, CJ: Changji, BoZ: Bozhou.

Despite their diverse geographic origins, the haplotype network of 163 *nad4* haplotypes from *H. contortus* populations in Xinjiang, China, showed no significant genetic structure, consistent with low pairwise genetic differentiation (Figure 4). Fst values, ranging from −0.013 to 0.043, indicated minimal genetic differentiation among the 8 populations, reflecting high gene flow (Figure 7). Although most populations showed extremely low genetic differentiation, the pairwise comparison heatmap (Figure 7) indicated that the Kashi (KS) population exhibited relatively higher differentiation. Notably, KS was genetically closest to the Atushi (ATS) population, and as shown in Figure 1, these 2 populations are also geographically proximate, providing a reasonable explanation for the observed genetic pattern.

AMOVA of 8 *H. contortus* populations from Xinjiang, China, revealed that 91.83% of genetic variation occurred within populations, with only 0.24% attributed to differences among populations (Table 4).

**Table 4.** AMOVA for *nad4* gene sequences in 8 *H. contortus* populations from Xinjiang, China

Source of variation	Degrees of freedom (df)	Sum of squares	Variance components	Percentage of variation (%)	Fixation index
Among populations	7	54.460	0.01785 Va	8.17	0.00241
Within populations	163	1206.049	7.39908 Vb	91.83	
Total	170	1260.509	7.41693		

## Discussion

This study provides the first comprehensive analysis of the genetic diversity of *H. contortus* based on the *nad4* gene across 8 populations in Xinjiang, China. All populations exhibited high haplotype diversity (Yin et al., 2013). The haplotype diversity in our Xinjiang populations (Hd = 0.995–1.000) is comparable to the high levels of genetic diversity (Hd = 0.993–1.000) reported for other Chinese *H. contortus* populations. Nucleotide diversity (Pi: 0.02007–0.03145) indicates substantial genetic variation, consistent with thresholds for high diversity (Pi > 0.005, Hd > 0.5; Grant and Bowen, 1998). Low Fst values (−0.013 to 0.043) suggest minimal genetic differentiation and high gene flow among populations, aligning with previous studies on *H. contortus* in China (Yin et al., 2013, 2016; Zhang et al., 2016).

AMOVA revealed that 91.83% of genetic variation occurred within populations, with only 0.24% among populations, indicating a negligible impact of geographic isolation on genetic structure. These findings are consistent with global studies on *H. contortus* populations (Gharamah et al., 2012; Hussain et al., 2014; Shen et al., 2017; Pitaksakulrat et al., 2021; Mannan et al., 2023; Arsenopoulos et al., 2024).

Phylogenetic and haplotype network analyses of 163 *nad4* haplotypes revealed no distinct geographic clustering, suggesting extensive gene flow likely driven by human-mediated sheep migration. This high gene flow is consistent with the open farming systems and extensive animal transportation practices common in Xinjiang, which facilitate the movement of parasites across regions. These results align with studies in China, Bangladesh and Greece, which report high gene flow across *H. contortus* populations using various genetic markers (Yin et al., 2013; Arsenopoulos et al., 2020; Parvin et al., 2022; Kalule et al., 2023).

Mismatch distribution analysis indicated a multimodal pattern, suggesting stable population dynamics in Xinjiang, likely due to high environmental adaptability. Tajima’s D values (−1.711 to −0.862) suggest population expansion or negative selection, but high gene flow (evidenced by Fst and AMOVA) precludes significant subpopulation differentiation. These findings collectively highlight frequent gene flow, likely facilitated by host movement, maintaining a homogeneous genetic structure across populations.

However, the interpretation of pronounced gene flow should be tempered by the limitations of the study. The use of a single mitochondrial marker (*nad4*) may lack the resolution to discern fine-scale population structure or to fully distinguish contemporary gene flow from shared ancestral polymorphism. This is a recognized constraint of mitochondrial DNA in nematode population genetics, where its resolution is often lower compared to nuclear markers (Blouin, 2002). Therefore, while the observed genetic homogeneity is consistent with high gene flow, the potential influence of historical demographic processes cannot be ruled out. Future studies utilizing high-resolution nuclear markers, such as genome-wide Single Nucleotide Polymorphisms, will be essential to accurately quantify the extent of contemporary gene flow and elucidate the population dynamics of *H. contortus* in this region.

With no commercial vaccine available for haemonchosis, control of *H. contortus* relies heavily on anthelmintics such as albendazole, ivermectin and levamisole (Kotze and Prichard, 2016). However, widespread anthelmintic resistance exerts strong selective pressure on parasite populations (Besier et al., 2016). Specifically, the common use of albendazole and ivermectin in Xinjiang, where resistance has been documented (Wang et al., 2017), means that the high gene flow observed among our studied populations likely accelerates the regional dispersal of resistance alleles, promoting resistant genotypes (Kotze et al., 2014; Chaudhry et al., 2015). This underscores the urgent need to investigate anthelmintic resistance in *H. contortus* in Xinjiang.

These findings provide critical insights into the adaptive evolution and population dynamics of *H. contortus* in Xinjiang, highlighting the role of gene flow in reducing geographic differentiation and enabling rapid adaptation to selective pressures, such as anthelmintic use (Kaplan and Vidyashankar, 2012). This study establishes a foundation for developing region-specific control strategies to manage anthelmintic resistance and mitigate the economic impact of this parasite, while acknowledging that its reliance on a single mitochondrial gene (*nad4*) represents a limitation. Future whole-genome studies could provide finer-scale resolution of population structure and resistance mechanisms to further advance these control efforts.

## Conclusions

In this study represents the first detailed investigation of *H. contortus* genetic diversity across 8 regions in Xinjiang. Data from the *nad4* gene suggest that high gene flow minimizes genetic differentiation despite geographic separation, which could facilitate the spread of anthelmintic resistance. These insights, while noting the limitation of a single genetic marker, enhance our understanding of *H. contortus* population dynamics and support the development of targeted control measures, contributing to broader efforts in biodiversity conservation and evolutionary ecology.

## Data Availability

The datasets of this article are included within the manuscript.
